# Genetic polymorphisms of fecundity genes in Watish Sudanese desert sheep

**DOI:** 10.14202/vetworld.2020.614-621

**Published:** 2020-04-05

**Authors:** Sara E. Ibrahim Mohamed, Romaz M. Ahmed, Khaleel I. Z. Jawasreh, M. A. M. Salih, Dalia Mursi Abdelhalim, A. W. Abdelgadir, Md. T. Obeidat, L. M. A. Musa, Mohammed-Khair A. Ahmed

**Affiliations:** 1 Animal Production Research Centre, Animal Resources Research Corporation, Ministry of Animal Resources, Khartoum, Sudan; 2 Institute for Studies and Promotion of Animal Exports, University of Khartoum, Khartoum, Sudan; 3 Department of Animal Production, Faculty of Agriculture, Jordan University of Science and Technology, Irbid, Jordan; 4 Institute of Endemic Disease, University of Khartoum, Khartoum, Sudan; 5 Department of Genetics and Bioinformatics, Central Laboratory, Ministry of Higher education and Scientific Research, Khartoum, Sudan; 6 Department of Biochemistry and Molecular Biology, Faculty of Science and Technology, Al-Neelain University, Khartoum, Sudan; 7 Department of Genetics and Animal Breeding, Faculty of Animal Production, University of Khartoum, Sudan

**Keywords:** fecundity, genes, litter size, sheep, Watish

## Abstract

**Background and Aim:**

The Watish sheep is a strain of desert sheep of smaller size compared to other desert sheep ecotypes, and there is anecdotal evidence that it is endowed with high litter size. The present study was designed for screening for polymorphisms in the known fecundity genes (bone morphogenetic protein receptor type 1B A<G in exon 6, bone morphogenetic protein 15 (*BMP15*) (*FecX^B^*, *FecX^G^*, *FecX^H^*, and *FecX^I^*) in exon2, growth differentiation factor 9 (*GDF9*) – G1 in exon1 and G8 in exon2 and PRLG<A in intron2) and their association with litter size in Watish.

**Materials and Methods:**

The study involved 156 Watish ewes of 2-6 years of age, along with data on litter size in the first, second, and third parity from Sinnar state and contiguous Blue Nile State. Genomic DNA was isolated and genotyped using polymerase chain reaction-restriction fragment length polymorphism. Allele and genotype frequencies were calculated by direct counting. Chi-square test for goodness of fit was performed for agreement with Hardy-Weinberg expectations and association testing.

**Results:**

The results demonstrated that all individuals were non-carriers for the target mutations of *FecB*, *BMP15* (*FecX^B^, FecX^H^*, and *FecX^I^*), and *GDF9*-G8. With regard to the *GDF9*-G1 gene, the genotypic frequencies were 0.07% (G+) and 0.93% (++), in *FecX^G^* gene they were 0.993% (++) and 0.006% (B+), in *PRL* gene 0.516(++), 0.347(B+), and 0.137(BB). The Chi-square test showed a non-significant association between ewe’s type of birth and the detected mutations genotypes.

**Conclusion:**

These results preliminarily indicated that *GDF9*-G1, *BMP15* (*FecX^G^*), and *PRL* genes might have had some contribution for improving litter size in Watish Sudanese sheep. However, further studies using larger samples are needed to detect the effects of those mutations on Watish sheep litter size.

## Introduction

Sudan is well endowed with livestock resources. The main sheep type in Sudan is the desert sheep, which encompasses a number of subtypes. The desert sheep ecotype is the most predominant for export and local consumption. Its meat is in high demand for export because of the size of its carcass, and the quality of its mutton and lamb. Desert sheep never had the opportunity to express their real genetic potential in production and reproduction as a result of the nomadic or semi-nomadic systems under which it is raised. Watish sheep, a subtype of desert sheep, are mainly found between latitudes 10° and 11° N along the Blue Nile. They are mainly owned by nomadic and semi-nomadic societies. This breed is of smaller size compared to all other desert sheep subtypes, which means that its ewes will have low maintenance requirements and thereby reduced feed costs. This makes it a possible candidate to be used as a dam breed. In addition, there is anecdotal evidence that the breed has high prolificacy though no systematic study has been carried out on it.

Ovulation rate and litter size are heritably controlled by several genes with minor effects, and sometimes also by single genes with major effects, named fecundity (Fec) genes [1]. The ovulation rate in sheep has been observed to be significantly increased by mutations in a closely linked group of genes. Those genes are bone morphogenetic protein receptor type 1B (*BMPR-1B*), bone morphogenetic protein 15 (*BMP15*), and growth differentiation factor 9 (*GDF9*), which are all part of the ovary-derived transforming growth factor-β (TGFβ) superfamily [2]. These genes have an important effect on ovulation rate and litter size [3]. *BMPR-1B*, the Booroola gene, also known as activin receptor-like kinase 6 (ALK6) and *FecB*, was the first major gene identified for influencing prolificacy in sheep [4]. BMPRIB is located at the *FecB* locus in between the SPP1 and EGF genes [5] in the 6^th^ autosome of sheep. It is inherited as a single autosomal locus with an additive effect for ovulation rate. *BMP15* gene, also known as Fec^X^ [6], is an X-linked gene (FecX locus) of sheep belonging to the TGFβ family. Ten mutations, labeled *FecX^G^*, *FecX^H^*, *FecX^I^*, *FecX^L^*, *FecX^B^*, *FecX^R^*, *FecX^Gr^*, *FecT^T^*, *FecX^W^*, and *FecX^O^*, have been detected within the *BMP15* with similar phenotypes, i.e. homozygous carrier ewes are sterile [7] and heterozygous carriers show increased ovulation rate [8,9]. *GDF9* gene, also known as *Fec^G^*, is mapped to the 5^th^autosome of sheep and is expressed in oocytes from the primary stage of follicular development until ovulation [10] playing an important role in folliculogenesis [11]. The gene consists of 2 exons separated by 1126bp intron and encodes a propeptide containing 453 amino acid residues. The active mature peptide is 135 amino acids long. The Prolactin gene is an anterior pituitary hormone having close interaction with gonadotropin, i.e., the elevation in the secretion of prolactin is normally associated with the pronounced reduction in gonadotropin secretion that results in stages of infertility [12].

The present study was designed for screening polymorphisms in these four Fec genes (*BMPR-1B*, *BMP15*, *GDF*, and *PRL*) and to test their association with litter size in the Watish Sudanese Desert sheep.

## Materials and Methods

### Ethical approval

According to the Animals Use in Research Committee of Khartoum University, this study does not require any special approval.

### Sample collection

Venous jugular blood samples from 156, 2 to 6years old Watish ewes along with data on litter size in the first, second, and third parity were collected from Sinnar State and contiguous Blue Nile State. Ewes were divided into two groups, according to their average litter size; the first group consisted of those ewes which had singletons, the second group consisted of those which had multiple births. The collected blood samples were transferred to the laboratory using the cooling chain and stored at −20°C for further analysis.

### DNA isolation

Genomic DNA was isolated using Qiagen Commercial kits. The quality and quantity of the extracted DNA was checked by Nanodrop Spectrophotometer and agarose gel electrophoreses. DNA samples were adjusted to a concentration of 100-200ng/μL and exactly 1 μL of the DNA samples were used as templates for polymerase chain reaction (PCR).

### Single nucleotide polymorphism detection assays

The *BMPR1B* gene locus was analyzed, targeting 141bp fragment covering the sequence containing the A<G substitution at 746^th^position of the open reading frame (ORF) (rs418841713) in exon 6. Polymorphism evaluation was conducted using PCR amplification, according to the procedure proposed by Wang *etal*.[13]. Similarly, four polymorphisms in exon 2 in *Ovis aries BMP15* *(Fec*X^B^, *Fec*X^G^, *Fec*X^H^, an*d Fec*X^I^) were evaluated. The specific mutations are: Gto T nucleotide change of *Fec*X^B^ at position 1100bp, C<T substitution (rs425019156) at position 718 of ORF in *Fec*X^G^ gene, C<T substitution (rs413916687) at position 871 in *Fec*X^H^ gene, and T<A substitution (rs398521635) at position 896 of the cDNA coding region in *FecX^I^*. Polymorphisms evaluation was conducted using PCR amplification according to the procedure proposed by Hanrahan *et al*. [11] for *FecX^B^* and *FecX^G^* and Lassoued *et al*. [6] for *FecX^H^* and *FecX^I^*.

Two polymorphisms G1 (G860A))rs410123449) at position 260 in the coding region, and G8 (C1184T) of the cDNA at position 395 of the mature protein were detected in *GDF9* gene located in exon1 and exon2, respectively, of Watish breed samples which were amplified, according to the procedure proposed by Hanrahan *et al*. [11].

The polymorphism of *Ovis aries* Prolactin (*PRL*) gene was at the G<T substitution (rs424801898) in intron 2, which was amplified according to the procedure proposed by Chu *et al*. [14] and Chu *et al*.[15]. Primer sequences, restriction enzymes, expected size, and annealing temperatures are illustrated in Table-1. Each PCR reaction was made in 25 μL volume containing 1× PCR buffer (10 mM Tris-HCl, 50 mM KCl); 1 μLdNTPs (10 mM), and 1.25 μL MgCl_2(_50 mM). 1 U of Taq polymerase, 1 μL of each primer (10 μM), and 100-200ng/μL DNA, then the volume was completed to 25 μL using d.dH_2_O. PCR cycles included: Initial denaturation at 94°C for 5min followed by 30cycles of denaturation at 94°C for 30 s, annealing for 30 s (Table-1), extension at 72°C for 30 s, and a last cycle of extension at 72°C for 8min. The PCR product was kept at 4°C until further analysis.

**Table-1:** Primer sequences, annealing temperatures, and PCR product sizes of the targeted genes conducted under this study.

Gene	(Primers 5’®3’)	Annealing temperature	Restriction enzyme	Product size
*BMPR-IB*	F:GTCGCTATGGGGAAGTTTGGATG	62°C	*Ava*II	140 bp
*FecB*	R:CAAGATGTTTTCATGCCTCATCAACACGGTC			
*BMP15*	F:GCCTTCCTGTGTCCCTTATAAGTATGTTCCCCTTA	62°C	*Dde*I	153 bp
*FecX^B^*	R:TTCTTGGGAAACCTGAGCTAGC			
*BMP15*	F:CACTGTCTTCTTGTTACTGTATTTCAATGAGAC	66°C	*Hinf*1	141 bp
*FecX^G^*	F:GATGCAATACTGCCTGCTTG			
*BMP15*	F:TATTTCAATGACACTCAGAG	59°C	*Spe*I	240 bp
*FecXH*	R:GAGCAATGATCCAGTGATCCCA			
*BMP15*	F:GAAAGTAACCAGTGTTCCCTCCACCCTTTTCT	63°C	XbaI	150 bp
*FecX^I^*	R:CATGATTGGGAGAATTGAGACC			
*GDF9-*G1	F- GAAGACTGGTATGGGGAAATG	58°C	*Hha*I	462 bp
	R- CCAATCTGCTCCTACACACCT			
*GDF9-*G8	F-CTTTAGTCAGCTGAAGTGGGACAAC	62°C	*Dde*I	139 bp
	R-ATGGATGATGTTCTGCACCATGGTG			
	TGAACCTGA			
*PRL*	F-ACCTCTCCTCGGAAATGTTCA	56°C	*Hae*III	1211 bp
	R-GGGACACTGAAGGACCAGAA			

The products of PCR were digested in a total of 10 μL reaction containing (10×) buffer 1 μL, 0.2 μL restriction enzyme, 5 μL PCR products, and 3.8 μL distilled water at a constant temperature of 37°C overnight in restriction fragment length polymorphism (RFLP) reaction. After 2% electrophoresis, the gel PCR products were visualized by a gel documentation system.

### Statistical analysis

Gene and genotypes frequencies were calculated by direct counting. AChi-square test for goodness-of-fit was performed to verify if genotype frequencies agreed with Hardy–Weinberg equilibrium expectations and for association testing.

## Results

### Genotyping of *BMPR-1B* gene

**Figure-1:**
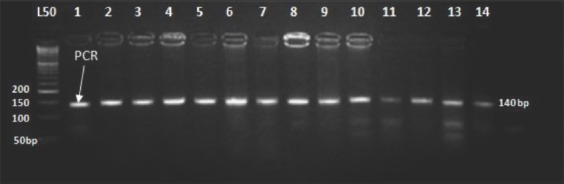
Agarose gel electrophoresis (4%) displaying *Ava*II1restriction site on an amplified portion of Watish *BMPR1B* gene in exon 6. Lane 1: Polymerase chain reaction product. Lane 2-14: Restriction enzyme representing BB genotype (140bp). Lane L: 50bp DNA ladder.

The missense mutation in *BMPR1B* gene causing substitution of A<G that results in a nonsynonymous substitution of glutamine with an arginine (Q249R) was not present in the studied populations. The digestion of the 140bp (PCR product) with *Ava*II (AG/CT) endonuclease enzyme resulted in one type of restriction pattern, which was assigned as homozygous wild type genotype (++) 140bp that produced one fragment (Figure-1). The allele and genotype frequencies of the *BMPR-1B* gene are shown in Table-2.

**Table-2:** Allele and genotype frequencies of the ovine *BMPR-IB*, *BMP15 (FecX^B^*, *FecX^G^*, *FecX*^H^, and *FecX*^I^), *GDF9* (G1-G8), and *PRL* genes in experimental population.

Gene	Genotype frequency			Allele frequency	
*BMPR-IB*	BB	D+	++	B	+
	0.00	0.00	1	0.00	1
*FecX^B^*	BB	B+	++	B	+
	0.00	0.00	1`	0.00	1
*FecX^G^*	GG	G+	++	G	+
	0.00	0.006	0.993	0.003	0.997
*FecX^H^*	CC	TC	TT	C	T
	0.00	0.00	1	0.00	1
*FecX^I^*	AA	TA	TT	A	T
	0.00	0.00	1	0.00	1
*GDF9*-G1	GG	G+	++	G	+
	0.00	0.07	0.93	0.036	0.964
*GDF9*-G8	GG	G+	++	G	+
	0.00	0.00	1	0.00	1
*PRL*	BB	B+	++	B	+
	0.137	0.347	0.516	0.31	0.69

### Genotyping of *BMP15* gene

**Figure-2:**
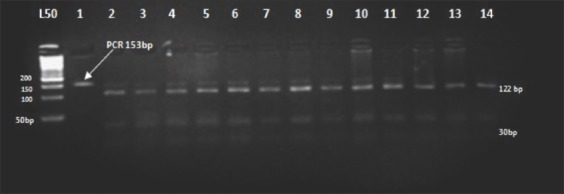
Agarose gel electrophoresis (4%) displaying *Dde*I1 restriction digest on an amplified portion of Watish *BMP15-FecX^B^* gene in exon 2. Lane: 1 polymerase chain reaction product. Lane: 2-14 representing ++ genotype (122-31bp). Lane L: 50 bpDNA ladder.

The *BMP15* (*FecX^B^*) locus was analyzed, targeting a 153bp fragment covering the sequence containing the missense mutation 1100 G <T(S99I) in exon 2 causing a non-synonymous substitution of serine with isoleucine. The PCR reaction was carried out with the restriction endonuclease enzyme *Dde*I (C/TTAG). It resulted in one type of restriction pattern that produced two fragments in all animals under study and was assigned as homozygous genotype (wild type) (122-31bp). There was a complete absence of both the homozygous genotype (mutant type) (153bp) and the heterozygous genotype (153-122 and 31bp) (Figure-2).

**Figure-3:**
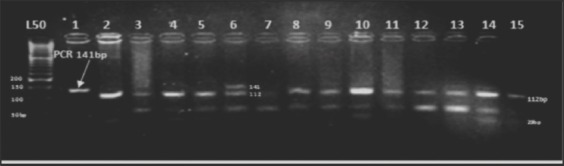
Agarose gel electrophoresis (4%) displaying *Hinf* I/digest an amplified portion of exon2 of the Watish *BMP15-FecX^G^* gene. Lane 1: Polymerase chain reaction product. Lanes 2-5, 7-15: Represent ++. Lane 6: Represent G+. Lane L: 50bp DNA Ladder.

The PCR product of *FecX^G^* was about 141bp, the non-sense mutation C<T which causes the substitution of glutamine with a stop codon (Q239*) resulting in premature termination of translation was detected by digestion with *Hin*f1I (G/ACT) endonuclease enzyme, generating two types of restriction patterns (112bp + 29bp) and (141bp + 112bp + 29bp) representing ++ and G+ genotypes, respectively. The wild type (++) was the more frequent type in the population, but none of the animals carried the homozygous mutant genotypeGG (141bp), which was not sensitive to the enzyme at the site of cutting (Figure-3).

**Figure-4:**
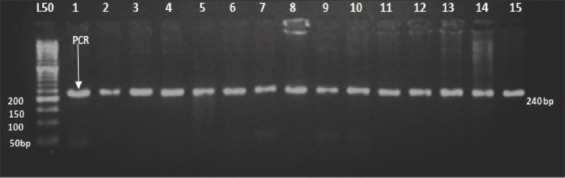
Agarose gel electrophoresis (4%) displaying *SpeI*/digest on an amplified product of Watish *BMP15-FecX^H^* in exon2. Lane 1: Polymerase chain reaction product. Lanes 2-15: Represent ++ 240bp. Lane L: 100bp DNA Ladder.

The point mutation polymorphism C<T in the *FecX^H^* gene causes the substitution of glutamine with a stop codon. The PCR products (240bp) evaluation was carried out with *Spe*I (A/CTAGT) endonuclease enzyme. The uncut pattern (240bp) resulted in one type of restriction fragment pattern, which was assigned as wild type genotype ++ in all animals under study (Figure-4).

**Figure-5:**
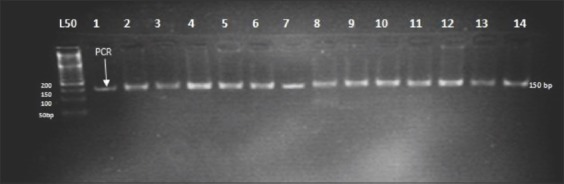
Agarose gel electrophoresis (4%) displaying *Xba*I digest in an amplified product of Watish *BMP15*-*FecXI* gene in exon 2. Lane 1: PCR product. Lanes 2-14: Represent ++ 150bp. Lane L: 50bp DNA Ladder.

For detection of the *FecX^I^* allele, 150bp of the PCR products covering the missense mutation polymorphism T<A was digested with the endonuclease restriction enzyme *Xba*I (T/CTAGA). This mutation results in nonsynonymous substitution of Valine with aspartic acid at amino acid position 308. Wild-type individuals for *FecXI* showed 150bp undigested fragments in all animals under study (Figure-5).

### Genotyping of *GDF9* gene

**Figure-6:**
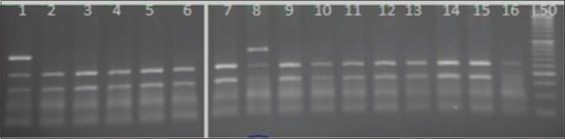
Agarose gel electrophoresis (4%) displaying *Hha*I digest on an amplified exon1of Watish *Fec^G^*-G1 gene. Lanes 2-7, 9-16: Represent++. Lanes 1, 8: (G+) lane L: 50bp DNA ladder.

The PCR product is about 462bp, located in exon 1 of the *GDF9*-G1, which included the transition mutation that changes adenine into guanine (G860A) at position 260 in the coding region. The mutation causes the substitution of amino acid arginine with histidine at residue 87. The product was digested with the endonuclease restriction enzyme *Hha*1 (GCG↑C). Digestion of the PCR product from wild-type*Fec^G^*(++) animals resulted in cleavage of the 462-bp product (at two internal *Hha*I sites) into fragments of 52, 156, and 254bp. The DNA fragments containing the A nucleotide gave only two fragments of 52 and 410bp (GG). Animals heterozygous (G+) for the mutation had fragments of all four sizes (52, 156, 254, and 410bp) (Figure-6).

**Figure-7:**
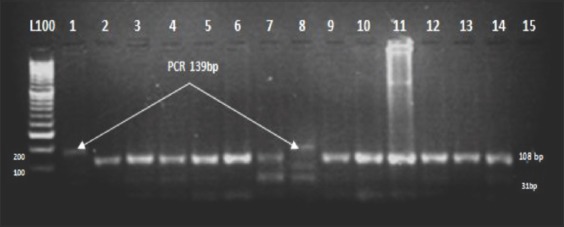
Agarose gel electrophoresis (4%) displaying *Dde*I/digest on an amplified exon 2 of Watish *Fec^G^*-G8. Lanes 1, 8: Polymerase chain reaction product (139bp). Lane 2-7, 9-15: Represent ++(108-31) bp. Lane L: 100bp DNA Ladder.

The transition mutation C<T in the *GDF9*-G8 causes the substitution of serine to phenylalanine at position 395 of the mature protein (Ser395Phe) in exon 2. Detection of the mutation in the 139bp PCR product in exon 2 of the *GDF9*-G8 that includes the mutation was carried out by digestion with the restriction endonuclease enzyme *Dde*I (C/TNAG). The digestion resulted in one type of restriction fragment pattern, which was assigned as wild type genotype ++: (108-31bp) and produced one fragment as displayed by the NEBcutter program in all animals under study. There was the complete absence of both homozygous (BB) genotype (139bp) and the heterozygous (B+) genotype (139-108 and 31bp) in all animals under study (Figure-7).

### Genotyping of *PRL* gene

**Figure-8:**
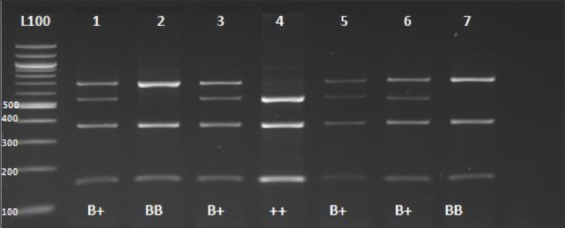
Agarose gel electrophoresis (2%) of the digested product of *PRL* gene at intron 2 by *Hae*III restriction enzyme. Lane L: DNA marker 100bp, Lane 1, 3, 5, and 6 heterozygous genotype. Lane 2 and 7 mutant type. Lane 4: Wild type genotype.

The digestion of the PCR product 1211bp located in intron 2, which includes the mutation causing the substitution of G<T, with endonuclease restriction enzyme *Hae*III (GG/CC) resulted in three types of restriction patterns: Homozygous genotypes which were assigned as wild type (++) (541, 372, 151, and 147bp), a mutant type (BB) (688, 372, and 151bp), and heterozygous genotype (B+) (688, 541, 372, 151, and 147bp) (Figure-8).

Allele and genotype frequencies calculated for all SNPs of genes under study are given in Table-2. The allele and genotype frequencies were calculated separately for ewes with single lambs, and those with more than one lamb (Table-3), and the frequencies were not significantly different in *BMPR*-IB, *BMP15* (*FecX^B^*, *FecX^H^*, and *FecX^I^*), and *GDF9*-G8 genes between the two groups of ewes (Table-3). However, there were differences between the two groups in the frequencies of *FecX^G^* and *GDF9*-G1 and *PRL* genes, but the differences were not statistically significant. The calculated Chi-square values were 0.219 and 3.39 for the three genes, respectively. There were no significant associations between birth type and the genotypes of these mutations (p>0.05).

**Table-3:** Allele and genotype frequencies of single and multiple litter ewes of the ovine *BMPR-IB*, *BMP15* (*FecX^B^*, *FecX^G^*,*FecX*^H^, and *FecX*^I^), *GDF9* (G1-G8), and *PRL* genes in the experimental population.

Gene	Single lamb ewes					Multiple litter ewes				
	
	Genotype frequency			Allele frequency		Genotype frequency			Allele frequency	
*BMPR-IB*	BB	D+	++	B	+	BB	D+	++	B	+
	0.00	0.00	1	0.00	1	0.00	0.00	1	0.00	1
*FecX^B^*	BB	B+	++	B	+	BB	B+	++	B	+
	0.00	0.00	1	0.00	1	0.00	0.00	1	0.00	1
*FecX^G^*	GG	G+	++	G	+	GG	G+	++	G	+
	0.00	0.00	1	0.00	1	0.00	0.008	0.992	0.004	0.996
*FecX^H^*	CC	TC	TT	C	T	CC	TC	TT	C	T
	0.00	0.00	1	0.00	1	0.00	0.00	1	0.00	1
*FecX^I^*	AA	TA	TT	A	T	AA	TA	TT	A	T
	0.00	0.00	1	0.00	1	0.00	0.00	1	0.00	1
*GDF9-G1*	GG	G+	++	G	+	GG	G+	++	G	+
	0.00	0.14	0.86	0.07	0.93	0.00	0.05	0.95	0.03	0.97
*GDF9-G8*	GG	G+	++	G	+	GG	G+	++	G	+
	0.00	0.00	1	0.00	1	0.00	0.00	1	0.00	1
*PRL*	BB	B+	++	B	+	BB	B+	++	B	+
	0.121	0.364	0.515	0.30	0.70	0.145	0.339	0.516	0.31	0.69

## Discussion

The Watish ecotype is an important genetic resource of Sudan. It is also different from other desert ecotypes in being of smaller size, of relatively high prolificacy and has good meat quality. This makes it a good candidate for use as a dam breed in a more specialized production system. Such a line may be used in crossbreeding with males from large ecotypes such as the Hamari and Kabashi.

SNPs sites in *BMPR*-IB, *BMP15*, *GDF9*, and *PRL* genes, which could affect litter size, were tested. The results showed that the Watish samples were homozygous for the 746 A allele (monomorphic for the glutamine variant) in the A>G SNP (Q249A, glutamine to an arginine). This indicates the absence of the Booroola mutation (*FecB*) in all studied animals and probably that it is not present in the Watish population at large. Similar results were found in other studies previously reported by Al-Barzinji and Othman [16], Nejhad and Ahmadi [17], and Abouheif *et al*. [18] in different sheep breeds in the Middle East.

Four previously reported mutations in exon 2 of the sheep *BMP-15* gene have been identified: *FecX^B^* (Belclare) (G<T), *Fec*X^G^ (Gallway) (C<T), *FecX^H^* (Hanna) (C<T), and *FecX^I^* (Inverdale) (T<A) were considered as candidate polymorphisms for the present study. No mutations in the *FecX^B^*, *FecX^H^*, and *FecX^I^* genes were found in Watish samples. Consequently, based on the evidence from this study, these mutations cannot be considered a cause of the anecdotal high prolificacy of Watish sheep. This suggests that the molecular mechanism affecting the multiparous performance in Watish sheep may be different from those of Inverdale, Hanna, and Belclare sheep. These results are in agreement with the results from Chios sheep [19], Hu sheep, and Chinese Merino [20].

The nonsense mutation in *FecX^G^* (C<T) at the position 718 leads to a premature stop codon at amino acid 239 of the unprocessed protein. The PCR product digested with HinfI was 141bp. The digestion demonstrated the existence of two patterns: The homozygous wild type *++* genotype (f=0.993) and a low frequency of heterozygous G+ genotype (0.006). The reason for the low frequency of the mutant allele is probably the fact that most Watish owners either slaughtered or sold infertile or barren ewes by the end of the 1^st^year of age. Similarly, Chu *et al*. [15] reported the absence of GG mutant genotype in Small-Tailed Han sheep. Ewes heterozygous for any one of these *BMP-15* mutations have experienced increased ovulation rates, whereas homozygous ewes were sterile due to a failure of normal ovarian follicular development [6,11].

The results of PCR-RFLP also indicated that Watish ecotype showed the existence of the targeted SNP of the *GDF9*-G1detected using *Hha*I digestion enzyme. The reaction produced two types of bands of 52, 156, and 254bp indicating homozygous wild type animals (++), four bands of 52, 156, 254, and 410bp for the heterozygous genotype (G+). The frequency of the wild type allele was 0.964, while the frequency of the mutant (G) allele was 0.036. The homozygous genotype (GG), which should produce 52 and 410bp bands as reported by Moradband *et al*. [21] in Baluchi breed and Hanrahan *et al*. [11] in Belclare and Cambridge breeds, did not appear in our Watish samples. Furthermore, Liandris *et al*. [22] did not find the mutant genotype in Karagouniki sheep.

The C/T mutation at 1184bp of *GDF9*–G8 leads to the change of Serine at amino acid 77 into Phenylalanine. All animals showed the same banding pattern of 139 pb in agreement with previously published results [23].

The PCR-RFLP amplification of the Prolactin gene (1211bp) showed that all three genotypes were present in our population. The allelic frequencies were 0.31 for the mutant B allele and 0.69 for the wild type allele (+), and the genotypic frequencies were 0.137 for BB, 0.347 for B+, and 0.516 for ++. These results are also congruent with reports in Awassi sheep in which the allelic frequencies were 0.757 for A allele and 0.243 for B allele, and the genotypic frequencies were 0.646 for AA, 0.221 for AB, and 0.131 for BB[24]. The results showed that prolificacy seemed to be affected by Prolactin gene variants. In Romanov sheep breed, Jawasreh *et al*. [25] reported the homozygous individuals (MM genotype) of GDF9 gene to produce 0.792 more lambs born per lambing than that of heterozygous NM genotype.

## Conclusion

No mutations in the *FecX^B^*, *FecX^H^*, and *FecX^I^* genes were found in Watish samples. Consequently, based on the evidence from this study, these mutations cannot be considered a cause of the anecdotal high prolificacy of Watish sheep. The Prolactin, *GDF9* (G1) and *FecX^G^* genes, showed variation but no association with litter size was detected. However, further studies using larger samples are needed to confirm or reject these results.

## Authors’ Contributions

MAA supervised, designed, and coordinated the study. SEIM collected samples, executed genotyping methods, analyzed genotyping data, and wrote significant parts of the manuscript. RMA wrote significant parts of the manuscript and coordinated the study. KIZJ contributed to data analysis, and contributed to the genotyping process reading and revising the manuscript. LMAM analyzed the data and coordinated the study. MAMS, DMA, and AWA contributed to laboratory work. MTO contributed in critical review and correction of the manuscript. All authors read and approved the final manuscript.
